# Assessing factors associated with one-year antibody waning in participants with repeated influenza vaccinations: A six-year cohort study

**DOI:** 10.1016/j.vaccine.2025.127904

**Published:** 2025-11-06

**Authors:** Cong Cheng, Xianyan Chen, Yang Ge, Meng-Hsuan Sung, Yuchen Zhang, Kehinde Ogunyemi, Michael A. Carlock, Hannah B. Hanley, Andreas Handel, Ted M. Ross, Ye Shen

**Affiliations:** a Department of Statistics, Franklin College of Art and Science, University of Georgia, Athens, GA, United States; b Department of Epidemiology and Biostatistics, College of Public Health, University of Georgia, Athens, GA, United States; c Center for Vaccines and Immunology, University of Georgia, Athens, GA, United States; d Department of Infectious Diseases, University of Georgia, Athens, GA, United States; e Florida Research and Innovation Center, Cleveland Clinic, Port Saint Lucie, Florida, United States; f Department of Infection Biology, Lerner Research Institute, Cleveland Clinic, Cleveland, OH, United States; g Center for the Ecology of Infectious Diseases, University of Georgia, Athens, GA, United States

**Keywords:** Influenza vaccine, Hemagglutination inhibition assay, Antibody waning, Repeat vaccination, Multiyear cohort

## Abstract

**Background:**

The long-term effects of host factors on vaccine-induced immune responses have been widely studied, but limited research has explored the waning of antibody levels after repeated influenza vaccinations in longitudinal settings.

**Methods:**

This study included 330 participants (584 observations), who received the standard-dose influenza vaccine across multiple seasons (≥2) from 2016 to 2022. Host demographics were collected prospectively, and serum samples were obtained pre- and post-vaccination each season. Linear mixed-effects models using restricted maximum likelihood (REML) were applied to evaluate waning scores, defined as the rate of decline in hemagglutination inhibition (HAI) composite scores, while accounting for demographic factors.

**Results:**

Higher BMI was significantly associated with greater antibody decline in the teenager subgroup, whereas no significant association was observed in adults or the elderly. In teenagers, age was negatively associated with antibody decline, whereas in adults (19–64 years), age was positively associated. Higher post-vaccination titers and greater pre-post vaccination boost scores were linked to increased antibody waning across all age groups, with significant effects observed in teenagers and adults but not the elderly. The differences by study influenza season influenced antibody waning in adults and the overall cohort but showed no significant effect in teenagers or the elderly (65+ years). Sex was not significantly associated with antibody waning in any age group.

**Conclusions:**

This study highlights multiple factors, including age, BMI, vaccination history, and immune response characteristics, that contribute to the waning of antibodies after repeated influenza vaccinations. These findings underscore the importance of personalized vaccination strategies to maintain long-term immunity and enhance vaccine effectiveness.

## 1. Introduction

Influenza virus infections pose a persistent public health burden, resulting in an estimated 120,000 to 700,000 hospitalizations and 12,000 to 51,000 deaths annually in the United States (U.S.) from the 2010–2011 to 2019–2020 influenza season [1,2]. Annual influenza vaccination remains a cornerstone for mitigating the spread of influenza and reducing severe outcomes. However, despite the widespread vaccine availability and strong recommendations for annual vaccination, vaccine effectiveness (VE) fluctuated significantly, ranging from 19 % to 60 % between the 2009–2010 and 2019–2020 influenza seasons [3-5]. These variations are attributed to several factors, including the rapid evolution of the virus and the potential for antigenic mismatch between the vaccine strains and the circulating viruses [6]. Even in seasons when the vaccine is well-matched, immunity acquired through vaccination is not long-lasting and can wane, leaving individuals susceptible to infections later in the season. Waning immunity is further influenced by host-related factors, such as immunosenescence, where immune responsiveness reduces with age [7-9].

Antibodies targeting the hemagglutinin (HA) protein of the influenza virus are well-recognized as key indicators of immunity to influenza [10]. Following infection or vaccination, antibody levels typically increase and then gradually diminish over time [11-13]. While factors influencing the rise have been extensively studied [3,4,14,15], the mechanisms and variability in the decline following annual vaccination remain areas needing further exploration. Understanding this decline of antibody titers, particularly in individuals undergoing repeated annual vaccinations, remain underexplored. This gap in understanding is crucial, as diminishing antibody levels affect the duration of vaccine-induced protection. European studies, following the 2011–2012 influenza season, reported that VE waned later in the season, leaving individuals increasingly vulnerable to infection [16,17]. Similarly, several U.S. studies have also highlighted the dynamics of waning immunity following influenza vaccination. Belongia et al. reported reduced protection against influenza A(H3N2) virus over time in community cohorts in an observational study of VE during the 2007–2008 season [18]. Hu et al. further observed mid-season declines in vaccine protection among Department of Defense beneficiaries across multiple seasons, emphasizing the importance of timing vaccinations effectively to maintain protection throughout each season [19]. Doyon-Plourde et al. confirmed through a systematic review and meta-analysis that vaccine-induced immunity wanes within a single season, reinforcing the need for strategies to mitigate intra-seasonal waning [20]. Chung et al. tracked nine influenza seasons in Ontario, Canada, finding that waning protection varies between seasons and across demographic subgroups, highlighting the importance of demographic-specific strategies [21]. Domnich et al. similarly noted mid-season declines in effectiveness against the A (H3N2) virus, stressing the importance of developing more durable vaccines [22]. Despite these insights into intra-seasonal waning, the long-term impact of repeated vaccinations across multiple seasons remains inadequately explored, with studies yielding inconsistent results regarding whether sequential vaccinations enhance or diminish immune protection [23-27]. For instance, Zelner et al. applied a Bayesian hierarchical model to assess the effects of sequential A(H1N1)pdm09 vaccination within a longitudinal cohort of older children and adults from 2011 to 2016, finding waning immunity over multiple seasons [28]. However, this study focused exclusively on one influenza strain and did not comprehensively account for host factors such as age and sex, which are known to influence immune responses [6,7]. These findings highlight the importance of investigating both intra-seasonal and multi-year waning, considering key demographic differences to fully understand the implications of repeated vaccinations.

Our study addresses critical gaps by conducting a longitudinal analysis of antibody decline in individuals receiving repeated annual influenza vaccinations. We examined three age groups: teenagers (10–18 years), adults (19–64 years), and the elderly (≥65 years), to explore age-related differences in immune responses. To assess the rate of antibody decline, we introduced the Hemagglutination Inhibition (HAI) Composite Waning Score, which provides insights into how antibody levels change over time and their relationship with influenza immunity. Furthermore, our study explores two critical factors beyond demographic information—the ‘boost effect’, which reflects the antibody increase following vaccination, and the ‘post-vaccination effects’, which measure how well antibody responses align with vaccine strains—in relation to the waning score. By utilizing data from a six-year cohort [29], we analyzed how these factors influence antibody waning to inform strategies for optimizing influenza vaccination, particularly for vulnerable populations like the elderly.

## 2. Methods

### 2.1. Study design

An ongoing human cohort study focusing on influenza vaccination was initiated during the 2016–2017 Northern Hemisphere influenza season in Athens, Georgia (GA), U.S., as previously described [4,29,30]. This study is ongoing and will continue at least through 2025. We used data from the 2016–2017 season to the 2021–2022 season, which were well-collected and organized. Participants were categorized into three age groups based on their age at the time of vaccination: teenagers (10–18 years), adults (18–64 years), and the elderly (65–85 years). The teenage cohort was incorporated starting from the second season.

In each influenza season, participants were enrolled and administered the Fluzone^™^ (Sanofi Pasteur, Swiftwater, PA, U.S.) split-inactivated influenza vaccine (IIV). Serum samples were collected at two timepoints: pre-vaccination (Day 0) and post-vaccination (Day 21/ 28) for each participant across all seasons. Post-vaccination samples were initially collected on Day 21 during the first two years (2016–17 and 2017–18), with the subsequent years shifting collection to Day 28, though actual collection days often varied slightly from these targets. Essential host factors, including age, sex, body mass index (BMI), and vaccination history, were systematically recorded upon re-enrollment each year. Vaccination history for the initial season (2016–17) was determined via self-reporting, while in subsequent seasons, a combination of self-reports with study records was used to ensure data accuracy.

To analyze trends in the waning of vaccine-induced immunity, the data were organized into five distinct waning assessment periods: 2016/17–2017/18, 2017/18–2018/19, 2018/19–2019/20, 2019/2020–2020/21, and 2020/21–2021/22. Participants with potential influenza infection, indicated by negative waning scores, were excluded. Our waning score model utilizes serum sample data, specifically, the Day 21/28 post-vaccination levels from the preceding year and Day 0 pre-vaccination levels in the subsequent year, for each waning assessment period under consideration.

#### Data Collection.

During each influenza season, enrollment began annually in September for eligible individuals who had not yet received the seasonal influenza vaccination. Recruitment took place at the University of Georgia (UGA) Clinical Trials Research Unit (CTRU) in Athens, GA, U.S., where participants provided written informed consent [4,29,30]. Serum samples were collected at two key time points for each participant: pre-vaccination (Day 0) and post-vaccination (Day 21/28). These samples were assessed for hemagglutination inhibition (HAI) titers in response to each influenza strain included in the vaccine. The influenza vaccine composition consisted of four virus subtypes: H1N1 and H3N2 (Influenza A), and Yamagata and Victoria (Influenza B). The full list of strains included in the vaccines during the six seasons, from 2016 to 17 to 2021–22, is available in Table 3.

Our analysis focuses exclusively on participants who received the standard-dose Fluzone^™^ inactivated influenza vaccine (IIV), manufactured by Sanofi Pasteur (Swiftwater, PA, U.S.), and participated in the study for at least two consecutive seasons between 2016 and 17 and 2021–22. This requirement ensures that the waning score capturing the decline in antibody titers over consecutive years could be accurately computed. Although elderly participants (65 years and older) could choose between high-dose and standard-dose vaccines, only data from those who received the standard-dose vaccine were included in the final analysis.

The dataset for this analysis consisted of 584 records from 330 participants, with each participant enrolled in at least one waning assessment period spanning two successive seasons. Demographic data collected for each participant included age, BMI, sex, years of participation, and vaccination history. Age groups were classified as teenagers (under 18 years), adults (18 to 64 years), and the elderly (65 years and older). Also, participants were categorized into normal (BMI < 25), overweight (BMI 25–30), and obese (BMI ≥ 30) groups based on their last BMI measure. Years of participation represented the cumulative number of seasons a participant stayed in the study until the current waning assessment period. Vaccination history was created as an indicator of whether a participant had received the influenza vaccine the year before the waning assessment period. We also created two new variables, Post-Vaccination Titer and Boost Composite Score, as explained below.

### 2.2. HAI composite waning score

To evaluate the decline of antibody responses across the four strains included in the Quadrivalent Influenza Vaccine (QIV), we develop a novel HAI composite waning score. This score reflects the sum of antibody decay rates from $t_{1}$ (post-vaccination) to $t_{2}$ (pre-vaccination of the subsequent year) across strains contained in the previous year’s vaccine, serving as the primary outcome measure. Typically, $t_{1}$ corresponds to Day 21/28 post-vaccination in the previous year, considered close to the peak antibody level, while $t_{2}$ corresponds to Day 0 before vaccination in the subsequent year. The formula for the waning score is as follows:

$$\text{Waning Score}=\sum_{s\in \{H1N1,H3N2,Bvic,Byam\}}\frac{\log\frac{HAI_{s}(t_{1})}{HAI_{s}(t_{2})}}{t_{2}-t_{1}}$$

where $HAI(t_{1})$ and $HAI(t_{2})$ are the HAI titers corresponding to a given influenza strain at time points $t_{1}$ and $t_{2}$, respectively, and $t_{2}-t_{1}$ represents the number of months between the two time points. Higher waning scores indicate greater antibody decay.

### 2.3. Post-vaccination titer and boost composite score

In addition to the waning score, we created two additional predictor variables: the post-vaccination titer and the boost composite score.

The post-vaccination titer measures the overall antibody levels induced by vaccination across all four strains in the previous year. It is calculated as the sum of log2-transformed HAI titers on Day 21/28 across all strains:

$$\text{Post}-\text{Vac}=\sum_{s\in \{H1N1,H3N2,Bvic,Byam\}}\log_{2}{\left(HAI_{post}\right)}_{s}$$

where $HAI_{post}$ refers to the HAI titers measured after vaccination. This measure serves as a surrogate for vaccine-induced protection.

The boost composite score evaluates the antibody response induced by the vaccine by comparing post-vaccination HAI titers (Day 21/28) with pre-vaccination titers (Day 0) within the same season. It is defined as the sum of the log2-transformed ratios of post- and pre-vaccination titers across all strains:

$$\text{Boost}=\sum_{s\in \{H1N1,H3N2,Bvic,Byam\}}\log_{2}{\left(\frac{HAI_{post}}{HAI_{pre}}\right)}_{s}$$

where $HAI_{pre}$ and $HAI_{post}$ are the pre- and post-vaccination HAI titers, respectively. This score approximates the antibody boost induced by the vaccine. To facilitate interpretation, the boost composite score was categorized into three levels: no boost (*Boost* ≤ 4), moderate boost (*Boost* > 4 and ≤ 8), and high boost (*Boost* > 8). This approach is from Abreu, R.B., et al. [30]. This categorization aligns with the log_2_-transformation of HAI titers, where serial twofold dilutions reflect antibody fold changes.

### 2.4. Statistical analysis

Descriptive statistics were calculated for the demographic characteristics of study participants, stratified by age group: teenagers (10–18 years), adults (18–64 years), and the elderly (65–85 years). These summaries provide an overview of the distribution of key variables, including age, BMI, sex, vaccination history, and years of participation. Scatter plots across age and BMI groups, with fitted linear regression lines and corresponding 95 % confidence intervals, were used to visualize the associations.

Linear mixed effect models with restricted maximum likelihood (REML) estimation were employed to examine the longitudinal association between host factors and waning scores. The mixed effect models were selected to account for repeated measures within individuals and to model individual-level variability not captured by the observed covariates. The models included random intercepts for each participant, accommodating correlations between repeated observations within individuals over time. The fixed effects in the models include: age group (teenagers, adults, elderly), sex, BMI, prior vaccination status (Yes/No), years of participation (ranging from 2 to 6), post-vaccination titer (Year 1), boost categories (Year 1), and the specific waning assessment period. Years of participation were quantified to range between 2 and 6, reflecting the number of seasons a participant remained in the study. A minimum value of 2 was necessary since participants needed to be enrolled for at least two consecutive years to complete one waning assessment period. Vaccination history was dichotomized as “Yes” or “No” depending on whether the participant received an influenza vaccine in preceding season. To address the potentially non-linear trends in the effect of time on HAI composite waning scores, dummy variables were introduced for each waning assessment period. The Satterthwaite approximation method was applied to determine the degrees of freedom for significance testing. We developed two levels of regression models: overall model included the entire study sample, accounting for variation across all age groups and host factors; and age-specific models for teenagers, adults, and the elderly, allowing for a more detailed exploration of age-related differences in waning immunity. Additionally, we conducted strain-specific analyses for each strain (H1N1, H3N2, Yamagata, and Victoria) included in the influenza vaccine, applying the same analytical framework as the main model but substituting the total score with the value for each specific strain. This approach ensured a comprehensive assessment of how each strain contributed to the overall waning score across different demographic groups. All statistical analyses were performed using R software version 4.3.2 [31]. The lmerTest package [32] was used to fit the mixed effect models and provide *p*-values for fixed effects, with the significance level set at *p* = 0.05. All reported p-values are nominal and have not been adjusted for multiple hypothesis testing.

### 2.5. Ethics statement

Participants were recruited at the UGA CTRU and the UGA Center for Vaccines and Immunology in Athens, GA, U.S. All participants enrolled provided written informed consent. The Institutional Review Board of the University of Georgia reviewed and approved the study procedures, informed consent, and data collection documents.

## 3. Results

### 3.1. Demographic data and waning score

Our study incorporated 584 blood specimens from 330 volunteers participating in the UGA cohort study over consecutive influenza seasons from September 2016 to March 2022; 218 observations with negative waning were excluded. Participant demographics, stratified by age groups and influenza seasons, are detailed in Table 1. The descriptive statistics for the scores created, including post-vaccination titer and boost composite score, and waning score are presented in Table 2. We also report the average of log2-transformed HAI titers for each influenza strain in Table S1. Notably, teenagers were not recruited during the 2016–2017 season, resulting in their absence from the 2016/17–2018/19 waning assessment period. Additionally, due to the exclusion of high-dose vaccine recipients in this analysis, elderly participants made up less than 5 % of the total sample size. The median BMI for adult and elderly participants was similar, ranging between 25.70 and 28.88 for adults, and 24.76 and 29.30 for elderly, indicating consistently elevated values in both groups. In contrast, teenagers had a lower median BMI, ranging from 21.16 to 23.01. There was a higher proportion of male participants among teenagers (41.7 % to 44.6 %) compared to adults (30.0 % to 38.2 %) (Table 1). However, chi-square tests of independence showed that these differences were not statistically significant (Table S2). Total vaccine-induced titer increases (boost composite score) were highest for the first year of the study than in the following years across different age groups (Table 2). Post-vaccination titers were predominantly higher in teenagers compared to the elderly and adults.

**Fig. 1.**
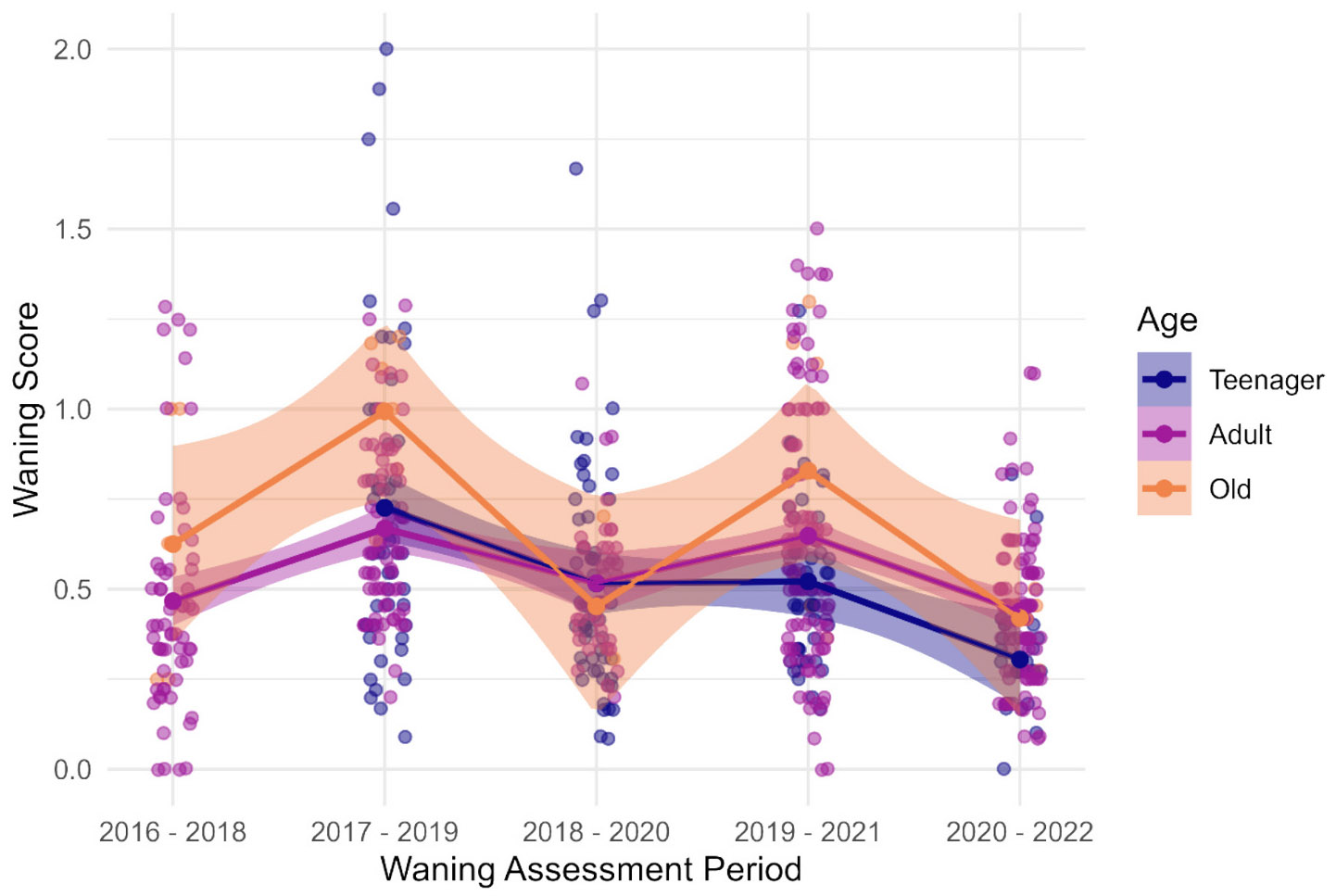
Mean waning scores with 95 % confidence interval by age groups over 5 waning assessment periods from 2016/17–2017/18 to 2020/21–2021/22. The waning scores were the summation of 2-fold changes among 4 influenza strains in the vaccine, and the age stratification was determined by the age in the last year of the waning assessment period for all individuals. Higher waning scores indicate greater antibody decay.

**Fig. 2.**
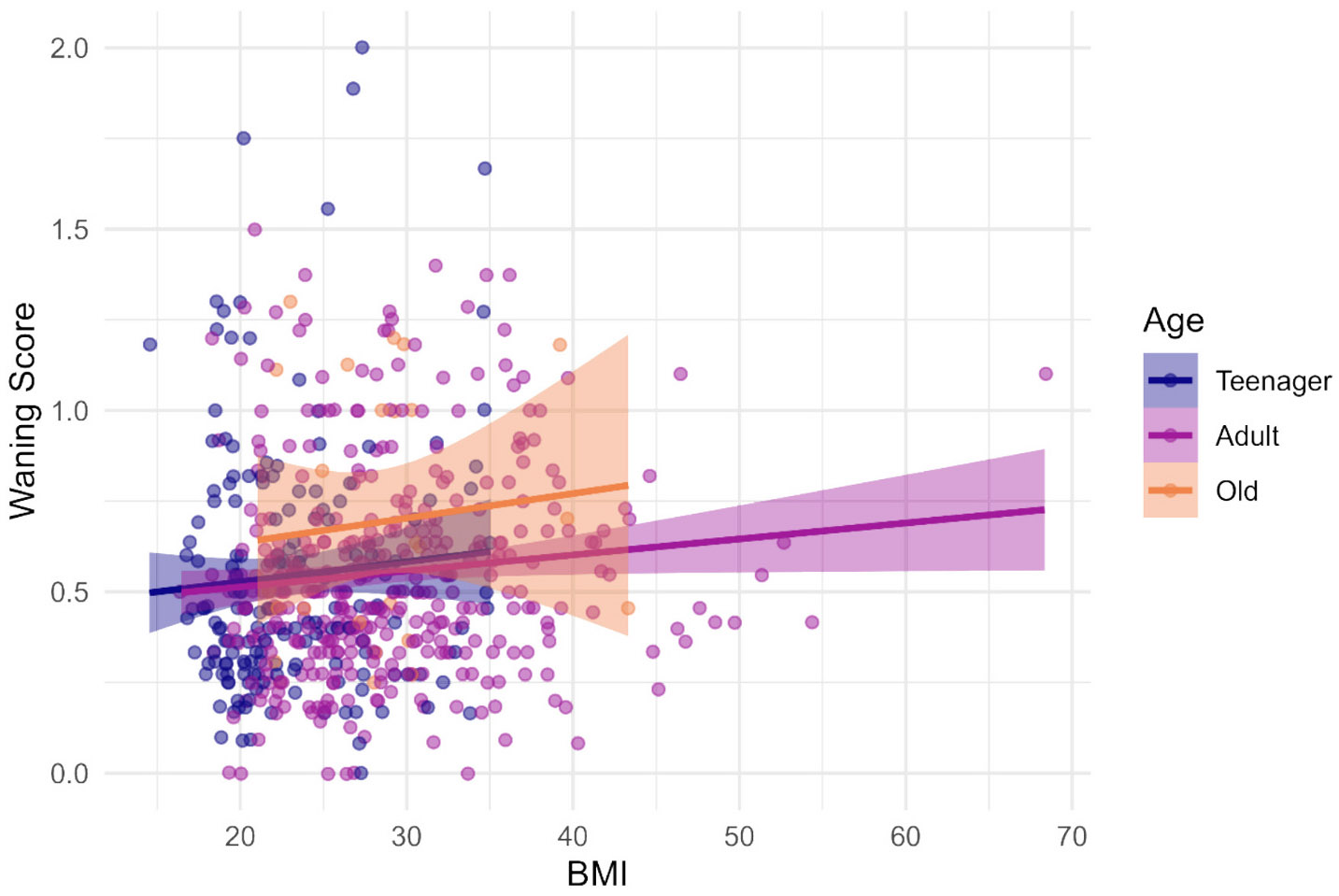
The linear trend with 95 % confidence interval between age and BMI to waning scores for all observations from waning assessment periods 2016/17–2017/18 to 2020/21–2021/22. The warning scores were the summation of 2-fold changes among 4 influenza strains in the vaccine, and the age stratification was determined by the age in the last year of the waning assessment period for all individuals. Higher waning scores indicate greater antibody decay.

**Fig. 3.**
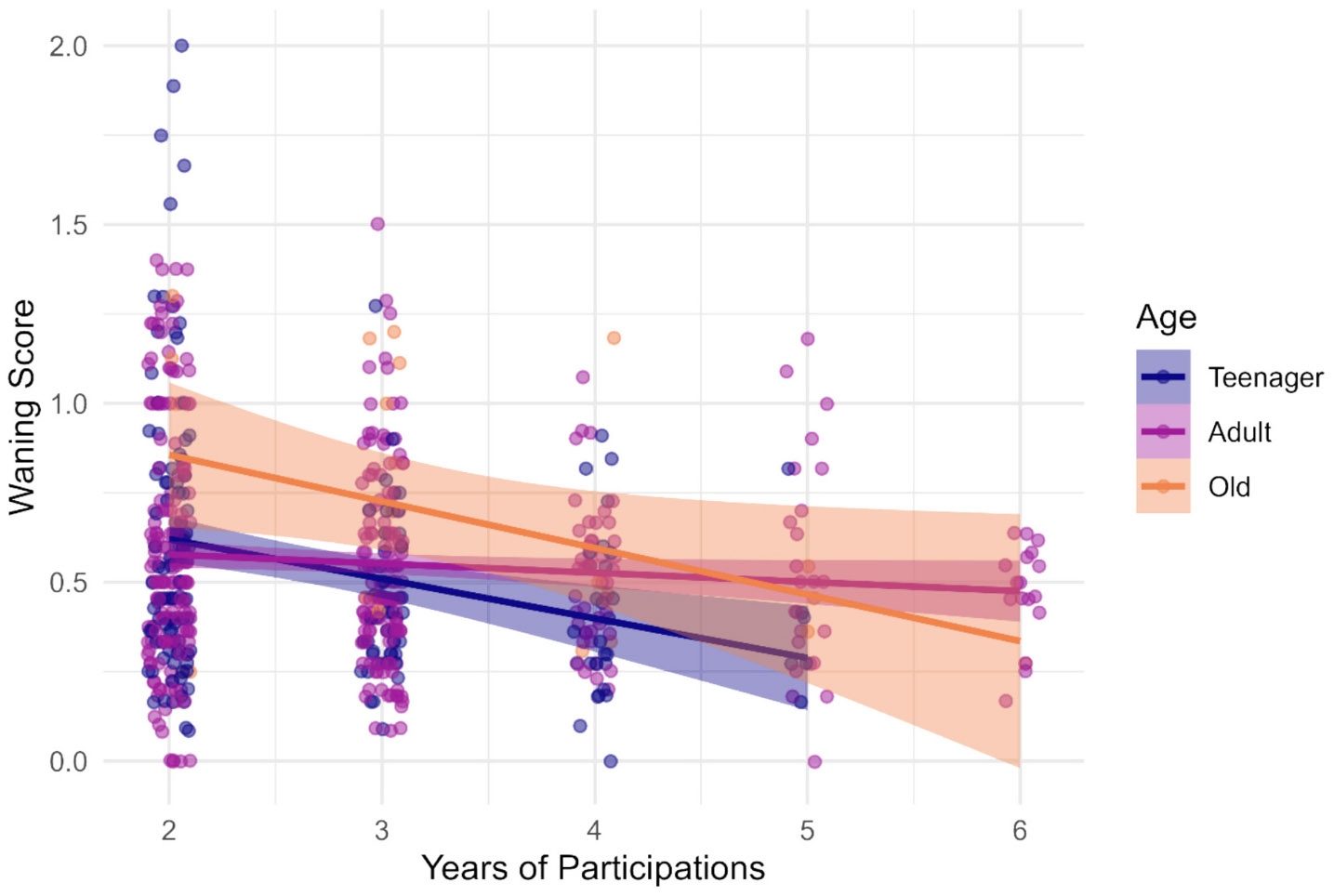
The linear trend with 95 % confidence interval between years of participation and age to waning scores. The definition of years in participations was following the time when HAIs were measured in an influenza season. The warning scores were the summation of 2-fold changes among 4 influenza strains in the vaccine, and the age stratification was determined by the age values in the last year of the waning assessment period for all individuals. Higher waning scores indicate greater antibody decay.

**Fig. 4.**
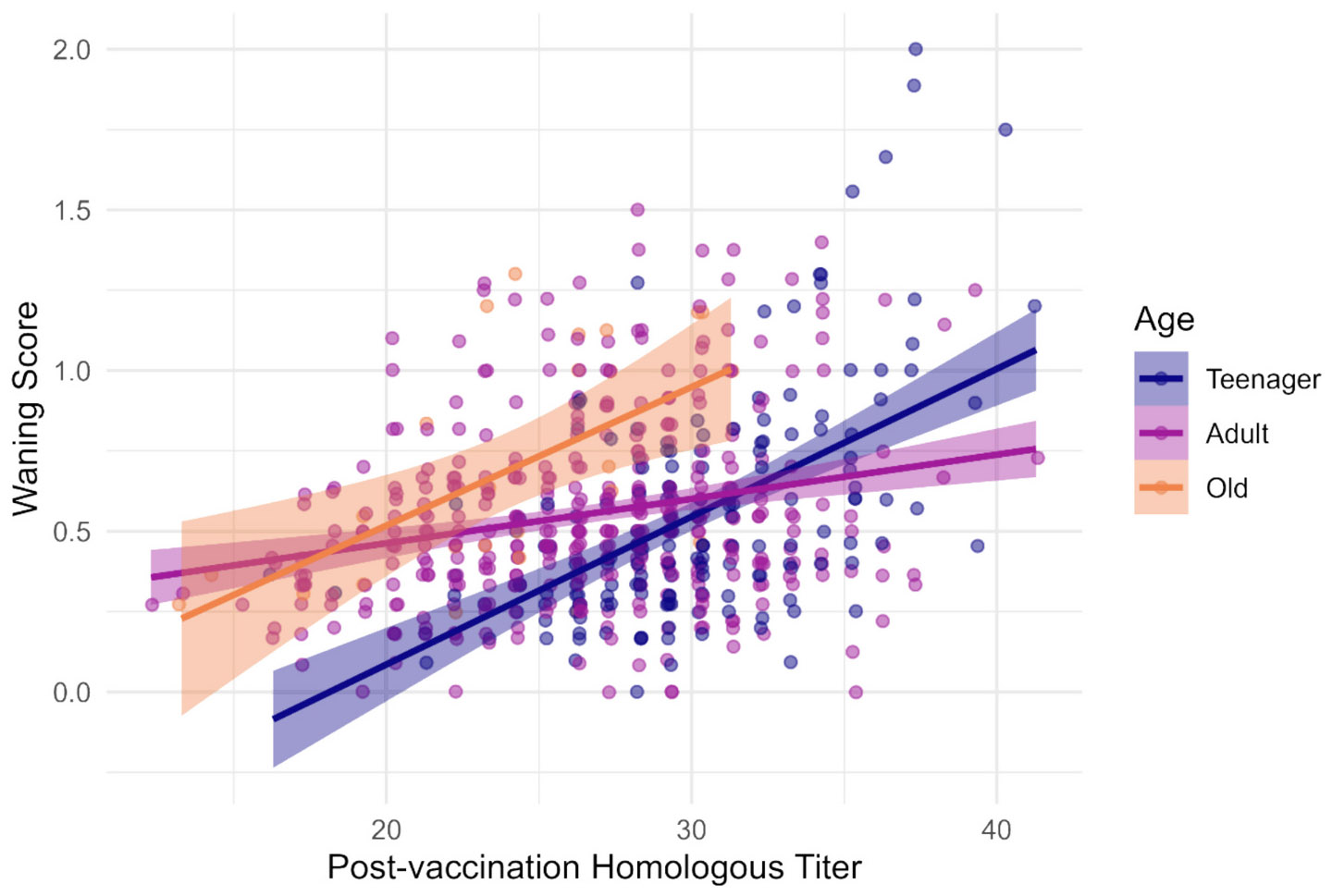
The linear trend with 95 % confidence interval between HAI and age to waning scores. The definition of post-vaccination titer was summation of geometric mean of HAI levels in the D21/28 in the previous year of the waning assessment period among 4 influenza strains in the vaccine. The waning scores were the summation of 2-fold changes among 4 influenza strains in the vaccine, and the age stratification was determined by the age values in the last year of the waning assessment period for all individuals. Higher waning scores indicate greater antibody decay.

**Fig. 5.**
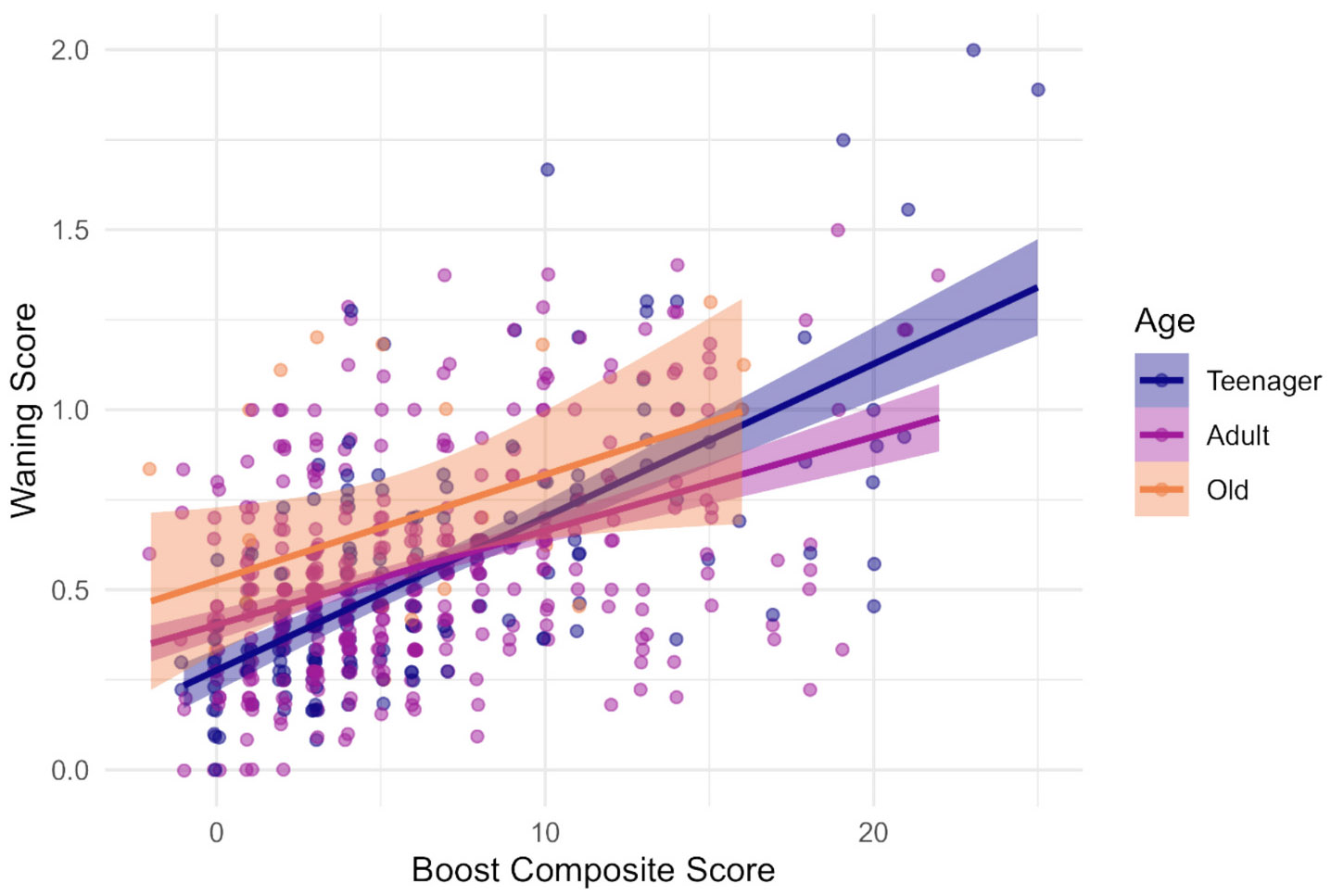
The linear trend with 95 % confidence interval between Boost and age to waning scores. The definition of boost composite score was the summation of fold changes between D0 and D21/28 in the previous year of the waning assessment period among 4 influenza strains in the vaccine. The warning scores were the summation of 2-fold changes among 4 influenza strains in the vaccine, and the age stratification was determined by the age in the last year of the waning assessment period for all individuals. Higher waning scores indicate greater antibody decay.

**Fig. 6.**
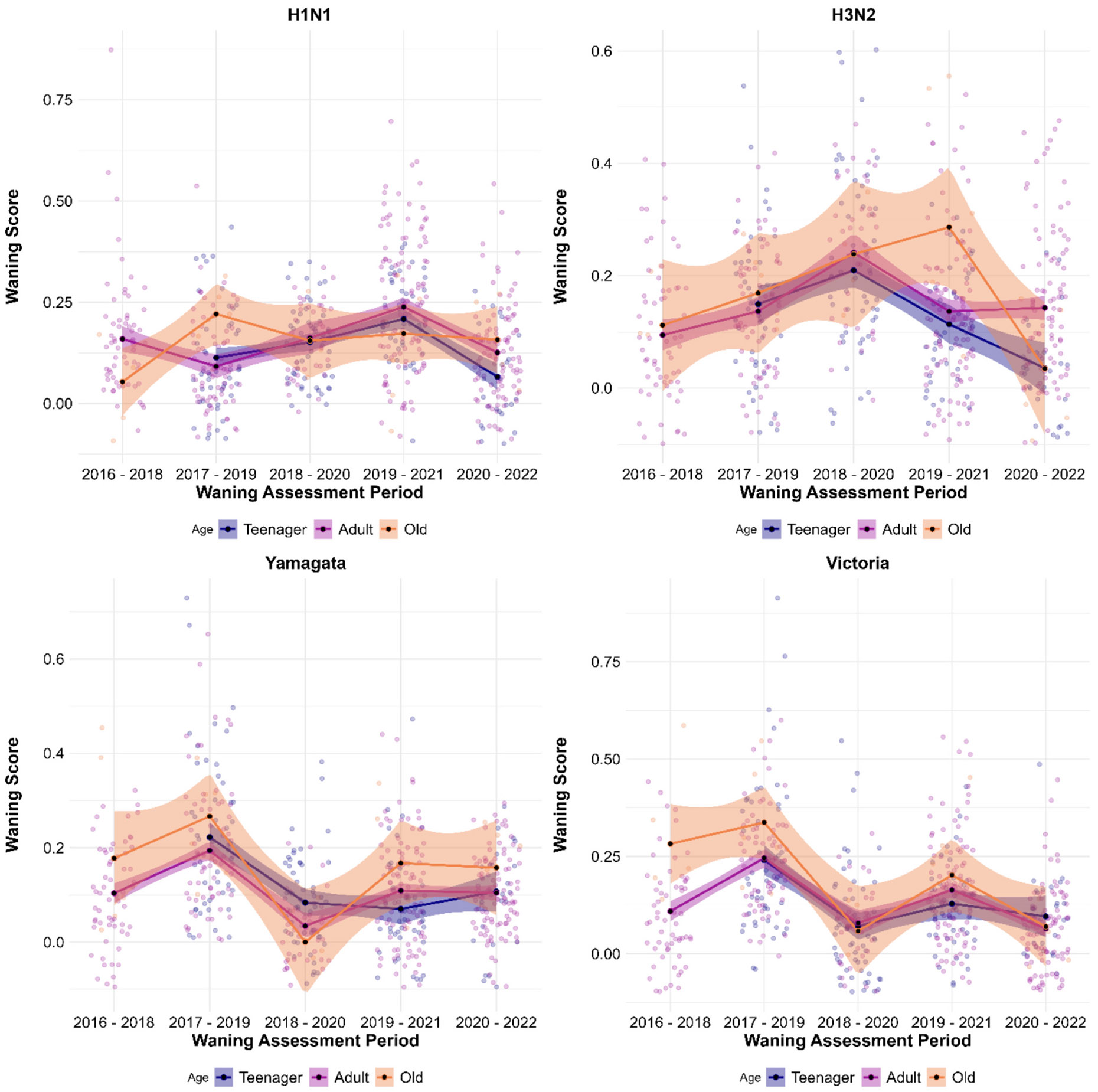
Mean waning scores with 95 % confidence interval by age groups over waning assessment periods for each influenza strain. Higher waning scores indicate greater antibody decay.

**Fig. 7.**
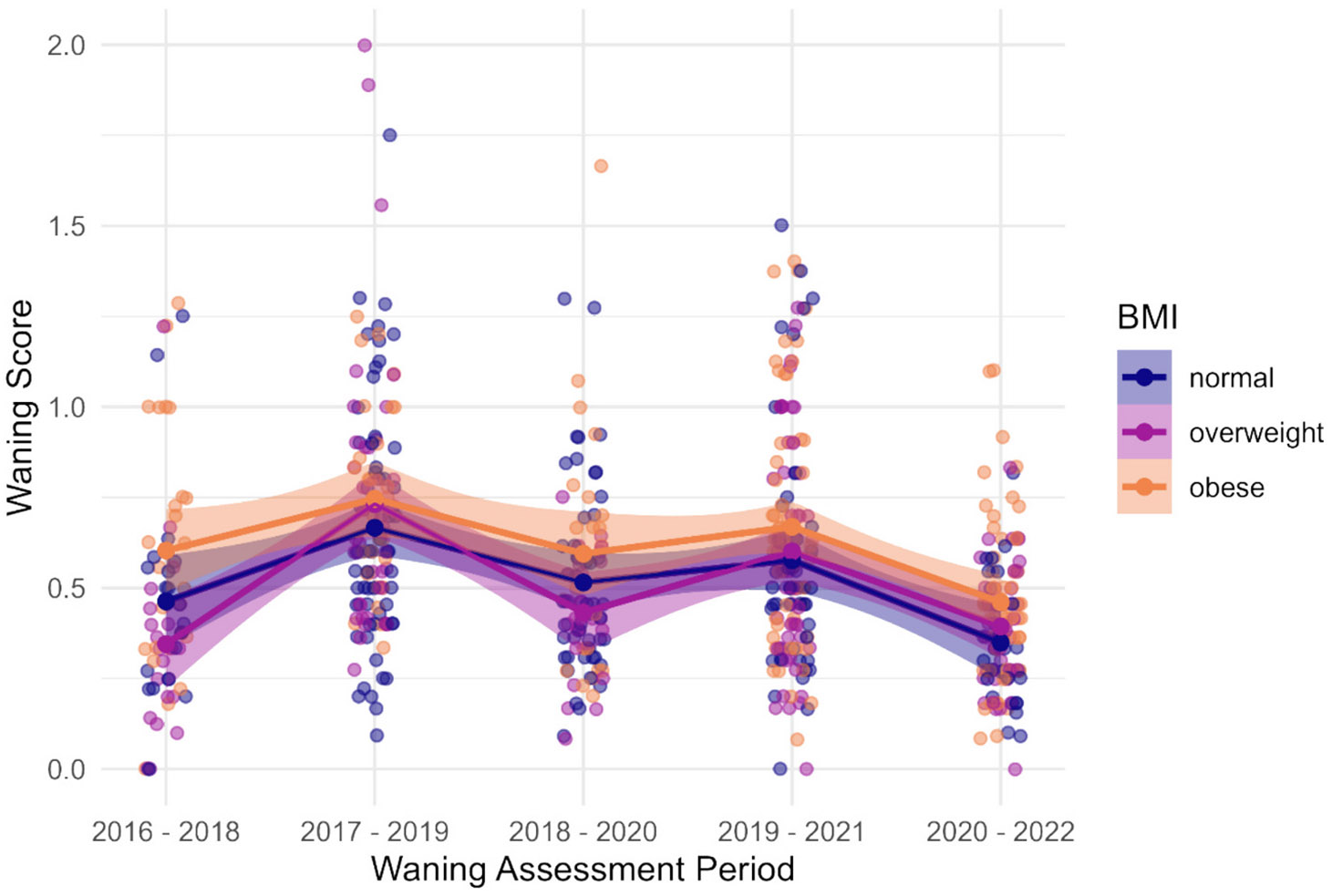
Mean waning scores with 95 % confidence interval by BMI groups over 5 waning assessment periods from 2016/17–2017/18 to 2020/21–2021/22. The waning scores were the summation of 2-fold changes among 4 influenza strains in the vaccine, and the BMI stratification was determined by the BMI values in the last year of the waning assessment period for all individuals. Higher waning scores indicate greater antibody decay.

Distinct patterns in waning scores across various host factors were evident. In Figs. 1-7, the trend lines represent univariate linear regression fits for each subgroup to aid visual interpretation. In Fig. 1, the average waning score for the elderly group shows two apparent rises around the 2017/18–2018/19 and 2019/20–2020/21 assessment periods, whereas the teenager and adult groups display a more gradual decline over time. Notably, all three groups showed an increase in mean waning scores as BMI increased (Fig. 2). Fig. 7 revealed variability in immunity waning across different BMI groups and time periods; period-specific ANOVAs across 2016–2018 to 2020–2022 yielded *p*-values of 0.0189, 0.493, 0.102, 0.294, and 0.025, respectively. Fig. 3 highlighted a negative correlation between years of participation and waning scores, with the relationship being stronger among old and teenage participants. Across all age groups, higher post-vaccination titers and boost composite scores were associated with increased waning scores, with the greatest trend observed in the elderly group (Figs. 4 and 5).

### 3.2. Modeling waning scores with host factors

A linear mixed-effects model was constructed to examine the relationship between waning scores and host factors, using all 584 observations. Significant positive correlations were observed between waning scores and factors such as age, BMI, influenza season, post-vaccination titers, and boost levels (Table 4 with estimation error and Table S3 with 95 % confidence interval). The positive coefficients associated with each waning assessment period indicator, though varying in magnitude, suggest increased waning responses to repeated influenza vaccination across assessment periods after 2016–2018, compared to the study’s initial year. Both moderate and high boost levels were associated with significantly higher waning scores compared to the no-boost group.

Subgroup analyses in Table 4, stratified by age group, revealed that the positive relationships between waning scores and post-vaccination titers remained consistent across teenagers, adults, and the elderly, though the association was weaker among elderly participants. A similar phenomenon was observed in the association between age and waning scores, which was also less pronounced among the elderly, likely due to a larger standard error resulting from the small sample size in this group. Sex differences were not significant across all age groups, and years of participation were also not significant. Period effects were less significant for teenagers, and only the 2017/18–2018/19 period showing significance for the elderly. In contrast, adults maintained consistent significance across all periods. The trend in boost levels persisted across teenagers and adults; yet among the elderly, there was no significant difference between the three levels’ boost categories, likely attributed to a limited sample size in this age group. We also conducted a sensitivity analysis by building the model with negative waning scores (Table S4), which yielded similar results.

### 3.3. Separate analyses by vaccine strain

Separate analyses of antibody decay rates for each vaccine strain revealed diverse patterns. The Yamagata and Victoria strains exhibited two peaks (period 2017–2019 and period 2019–2021) across the five waning assessment periods (Fig. 6), similar to the overall trend shown in Fig. 1. In contrast, the H1N1 and H3N2 strains displayed a single peak.

Notably, the elderly group showed peak waning at different time points compared to the teenage and adult groups. The relationship between waning scores and host factors for individual strains mirrored the patterns observed in the primary analysis across all strains (Table 5). Age and post-vaccination titers were consistently positively correlated with waning scores across all strains, aligning with findings from the primary analyses (Table 4). Similarly, boost levels showed significant positive coefficients across all strains except Victoria, indicating a consistent relationship between higher boost levels and increased waning. Interestingly, Yamagata showed a unique correlation (*p* = 0.051) with prior vaccination status, highlighting that time-dependent variability potentially impacts the immune response to this strain.

## 4. Discussions

In this six-year cohort study spanning from 2016 to 2022, we examined the relationship between waning scores and host factors across multiple waning assessment periods. Our analysis offers insights into how demographic and biological variables influence the decline in vaccine-induced antibody levels over time.

The linear mixed effect models showed significant positive associations between age, BMI, post-vaccination titers, waning assessment periods, and boost levels with waning scores (Table 4). Participants who were younger, had lower BMI, lower post-vaccination titers, or lower boost levels were less likely to experience rapid declines in antibody levels. Our findings suggest that age was significant in the model including all participants, confirming that age plays a critical role in antibody waning. This aligns with previous research showing that immunosenescence, the age-related decline in immune function, leads to weaker immune responses and faster antibody decay in older adults [30,33,36]. Importantly, in our study, the number of older adult participants was very small (4–6 per year), so our subgroup results should be interpreted as descriptive rather than conclusive. Nevertheless, placing these observations in the context of prior evidence highlighted the importance of considering tailored vaccination strategies for older adults, as they are more susceptible to waning immunity.

Similar to previous studies [33], which linked post vaccinations to the decline in HAI titers among community-dwelling adults aged 75 and older, our results also indicate that these factors are associated with a reduction in antibody titers across all age groups receiving repeated vaccinations. However, they reported less waning in individuals with higher post-vaccination HAI titers, which contrasts with our findings. This discrepancy may be due to their study population consisting of highly vaccinated older adults. Different waning assessment periods had varying influences on the waning scores, emphasizing the seasonal variability in vaccine response. The vaccination in the waning assessment periods 2020/21–2021/22 seem to result in a smaller waning compared to vaccinations from previous years. Furthermore, our results also provided insight into boost levels, with participants showing higher initial antibody responses experiencing faster decay. This suggests that while robust early responses reflect vaccine effectiveness, they may lead to quicker antibody loss. Notably, because the end-of-season titer is used both as the final point for waning in one season and as the baseline for boosting in the next, boosting and waning can be viewed as related measures—different sides of the same coin in annually vaccinated individuals at steady state.

Additionally, Wunderlich et al. suggested that sex had an impact on waning [33], while in our study, this factor had minimal influence. Across all age groups, sex was not significantly associated with waning scores in the mixed-effects model (Table 4), consistent with prior studies reporting limited or no sex-related differences in seasonal influenza vaccine responses [34-37].

Although BMI was statistically significant in our model, its per-unit effect on waning scores was modest. While it is well known that BMI significantly influences immune responses, the effect of obesity on the waning immune response to influenza vaccination remains complex and poorly understood across different influenza strains. Several studies have shown that BMI affects the waning of COVID-19 vaccination [38,39]. However, King et al. found that BMI was not significantly associated with reduced influenza vaccine effectiveness for all three influenza types/subtypes [A(H3N2), A(H1N1)pdm09, and B] [40].

A notable strength of our study lies in the utilization of linear mixed-effect models, which account for the correlations between repeated measurements across multiple waning assessment periods. This modeling approach allows for a robust analysis of long-term VE and the interaction of various host factors, providing valuable insights into the effects of host factors within different demographic groups.

However, several limitations should be acknowledged. First, serum samples were collected only once per flu season, limiting our ability to capture short-term variations in antibody levels. Second, the small number of elderly participants who received the standard-dose vaccine restricts the generalizability of our findings for this subgroup. Third, the lack of racial diversity within the cohort limits the applicability of our results to broader populations. Fourth, our study focused solely on humoral immune responses, excluding cell-mediated immunity, which is critical for a comprehensive immunological assessment. Fifth, although all HAI assays were conducted in the same laboratory using standardized protocols, the timing of sample processing was not recorded, and potential batch effects could not be assessed or adjusted for. Additionally, we must consider the possibility of residual confounding from unmeasured confounders such as comorbidities and influenza infection status.

In conclusion, our study highlights the complex interplay between host factors and antibody waning, emphasizing the need for targeted vaccination strategies. Individuals who are younger, have lower BMI, lower post-vaccination titers, and lower boost levels are less likely to experience a rapid decline in immunity. While our results for older adults are based on a very limited sample, they are consistent with prior evidence that age-related immune decline may accelerate waning [30,33,36]. These findings underscore the importance of tailored vaccination schedules and booster strategies, particularly for older adults and individuals with weaker immune responses, to maintain optimal vaccine efficacy throughout the influenza season. Further research with larger and more diverse elderly populations is necessary to improve vaccination policies and ensure better protection for at-risk groups across different demographics.

## Supplementary Material

1

## Figures and Tables

**Table 1:** Demographic statistics of subjects enrolled^1,2^.

	All	Teenagers	Adults	Elderly
**Sample size (%)**	584	172 (29.5 %)	386 (66.1 %)	26 (4.4 %)
**Yearly participation (%)**				
*2016/17–2017/18*	67 (11.5 %)	–	62 (92.5 %)	5 (7.5 %)
*2017/18–2018/19*	126 (21.6 %)	48 (38.1 %)	72 (57.1 %)	6 (4.8 %)
*2018/19–2019/20*	100 (17.1 %)	56 (56.0 %)	40 (40.0 %)	4 (4.0 %)
*2019/20–2020/21*	159 (27.2 %)	43 (27.0 %)	110 (69.2 %)	6 (3.8 %)
*2020/21–2021/22*	132 (22.6 %)	25 (18.9 %)	102 (77.3 %)	5 (3.8 %)
**Number of years participation (%)**				
*2*	266 (45.5 %)	94 (54.6 %)	165 (42.7 %)	7 (26.9 %)
*3*	189 (32.3 %)	45 (26.2 %)	134 (34.7 %)	10 (38.5 %)
*4*	78 (13.4 %)	27 (15.7 %)	46 (11.9 %)	5 (19.2 %)
*5*	33 (5.7 %)	6 (3.5 %)	24 (6.2 %)	3 (11.6 %)
*6*	18 (3.1 %)	–	17 (4.5 %)	1 (3.8 %)
**Sex-Male (%)**				
*16–18*	27 (40.2 %)	–	23 (37.1 %)	4 (80.0 %)
*17–19*	50 (39.7 %)	20 (41.7 %)	26 (36.1 %)	4 (66.7 %)
*18–20*	39 (39.0 %)	25 (44.6 %)	12 (30.0 %)	2 (50.0 %)
*19–21*	61 (38.4 %)	18 (41.9 %)	42 (38.2 %)	1 (16.7 %)
*20–22*	46 (34.8 %)	11 (44.0 %)	34 (33.3 %)	1 (20.0 %)
**Age-median (IQR)**				
*16–18*	27 (23, 45)	–	26 (23, 36.75)	67 (66, 69)
*17–19*	23.5 (16, 37)	15 (14, 16)	29 (25, 47.25)	67.5 (66.25, 72.5)
*18–20*	17 (15, 35)	15 (14, 16)	38 (26.75, 53)	65.5 (65, 66.5)
*19–21*	40 (17, 51.5)	15 (14.5, 16)	45 (36, 53)	68 (66.25, 69.75)
*20–22*	40.5 (25, 53)	16 (15, 17)	44 (32, 53.75)	69 (67, 71)
**BMI-median (IQR)**				
*16–18*	26.88 (23.48, 30.94)	–	26.70 (23.36, 31.16)	29.30 (28.13, 30.35)
*17–19*	24.88 (21.27, 28.52)	21.16 (19.61, 24.34)	26.91 (22.85, 30.61)	28.86 (25.82, 29.64)
*18–20*	23.54 (20.81, 28.34)	21.75 (19.58, 25.29)	27.52 (23.17, 32.25)	28.60 (26.64, 31.66)
*19–21*	27.24 (22.82, 31.73)	23.01 (20.04, 27.60)	28.64 (24.95, 32.99)	24.76 (22.36, 29.20)
*20–21*	27.66 (23.95, 33.48)	22.18 (19.16, 27.36)	28.88 (25.14, 34.75)	27.18 (23.76, 30.32)
**Prior Vaccination-Yes (%)**				
*16–18*	47 (70.1 %)	–	42 (67.7 %)	5 (100.0 %)
*17–19*	59 (46.8 %)	0 (0.00 %)	53 (73.6 %)	6 (100.0 %)
*18–20*	56 (56.0 %)	14 (25.0 %)	38 (95.0 %)	4 (100.0 %)
*19–21*	133 (83.6 %)	43 (100.0 %)	86 (78.2 %)	4 (66.7 %)
*20–22*	125 (94.7 %)	22 (88.0 %)	98 (96.1 %)	5 (100.0 %)

1 584 blood specimens from 330 volunteers participating in the UGA cohort study over consecutive influenza seasons from September 2016 to March 2022 were included.

2 Data are presented as count (%), or median (IQR), where IQR is the interquartile range (25th–75th percentile).

**Table 2:** Post-Vaccination Titer, Boost Composite Score, and waning score of participants enrolled. The definition of post-vaccination titer was summation of geometric mean of HAI levels in the D21/28 in the previous year of the waning assessment period among 4 influenza strains in the vaccine. The dfinition of boost composite score was the summation of 2-fold changes between D0 and D21/28 in the previous year of the waning assessment period among 4 influenza strains in the vaccine.^1^

	All	Teenagers	Adults	Elderly
**Post-Vac-median (IQR)**				
*16–18*	31.29 (28.29, 33.29)	–	31.29 (29.29, 34.04)	27.29 (26.29, 27.29)
*17–19*	31.29 (28.29, 32.29)	32.29 (31.29, 35.29)	29.29 (27.29, 32.29)	24.79 (23.29, 26.29)
*18–20*	28.29 (25.29, 30.29)	30.29 (27.29, 33.29)	25.79 (22.29, 28.54)	23.29 (18.79, 28.04)
*19–21*	26.29 (22.29, 29.29)	28.29 (26.29, 29.29)	24.29 (21.29, 28.29)	23.29 (20.04, 26.54)
*20–22*	25.29 (22.29, 27.54)	27.29 (26.29, 28.29)	24.29 (21.29, 27.29)	23.29 (22.29, 24.29)
**Boost-median (IQR)**				
*16–18*	10 (6, 15)	–	11 (6, 15)	7 (5, 10)
*17–19*	3 (1.25, 7)	8 (3, 14)	2 (1,3)	1.5 (1, 2.75)
*18–20*	4 (2, 6)	4 (3, 10.25)	3.5 (2, 5)	1 (0.75, 2.75)
*19–21*	5 (3, 9.5)	4 (2, 5)	7 (3.25, 10)	8 (5.25, 13.75)
*20–22*	4 (3, 7)	3 (2, 6)	4 (3, 7)	6 (5, 7)
**Number of boost categories (%)**				
*No-Boost (≤ 4)*	296 (50.7 %)	90 (30.4 %)	195 (65.9 %)	11 (3.7 %)
*Moderate-Boost (> 4 & ≤ 8)*	116 (19.9 %)	30 (25.9 %)	78 (67.2 %)	8 (6.9 %)
*High-Boost (> 8)*	172 (29.4 %)	52 (30.2 %)	113 (65.7 %)	7 (4.1 %)
**Waning score-median (IQR)**				
*16–18*	0.40 (0.26, 0.60)	–	0.40 (0.28, 0.57)	0.63 (0.28, 0.57)
*17–19*	0.63 (0.45, 0.89)	0.60 (0.40, 0.93)	0.61 (0.50, 0.83)	1.06 (0.88, 1.16)
*18–20*	0.46 (0.33, 0.62)	0.42 (0.30, 0.69)	0.52 (0.38, 0.62)	0.40 (0.33, 0.53)
*19–21*	0.55 (0.38, 0.82)	0.45 (0.33, 0.64)	0.60 (0.40, 0.90)	0.83 (0.48, 1.17)
*20–22*	0.40 (0.27, 0.50)	0.27 (0.18, 0.36)	0.44 (0.27, 0.55)	0.45 (0.42, 0.45)

1 Data are presented as count (%), or median (IQR), where IQR is the interquartile range (25th–75th percentile).

**Table 3:** Components of influenza vaccine used in UGA study between 2016 and 2022.

Year	Vaccine strains			
	A strains of influenza		B strains of influenza	
	H1N1	H3N2	Yamagata	Victoria
2016–2017	CA/09	HK/14	Phu/13	BR/08
2017–2018	MI/15	HK/14	Phu/13	BR/08
2018–2019	MI/15	Sing/16	Phu/13	CO/17
2019–2020	BR/18	KS/17	Phu/13	CO/17
2020–2021	GD/19	HK/19	Phu/13	WA/19
2021–2022	Vic/19	TAS/20	Phu/13	WA/19

(BR: Brisbane; CA: California; CO: Colorado; GD: Guangdong; HK: Hong Kong; KS: Kansas; MI: Michigan; Phu: Phuket; Sing: Singapore; TAS: Tasmania; Vic: Victoria; WA: Washington)

**Table 4:** Coefficient estimates (standard errors in parentheses; significance levels indicated by asterisk) of demographic factors from the linear mixed-effects model for overall samples and each age group.

	All (*n* = 584)	Teenagers (*n* = 172)	Adults (*n* = 386)	Elderly (*n* = 26)
Age^1^	**0.004** *** **(0.001)**	**−0.039** * **(0.017)**	**0.002** * **(0.001)**	−0.009 (0.017)
Sex (Male)	−0.019 (0.021)	0.023 (0.045)	−0.036 (0.025)	−0.091 (0.129)
BMI^2^	**0.004** * **(0.002)**	**0.012** * **(0.005)**	0.001 (0.001)	−0.002 (0.011)
Prior vaccination (Yes)	0.016 (0.029)	−0.102 (0.089)	−0.004 (0.033)	**−0.477^.^ (0.242)**
Number of years participation	−0.002 (0.013)	0.104 (0.061)	−0.006 (0.014)	0.120 (0.098)
Period 17–19	0.395*** **(0.040)**		0.417*** **(0.045)**	0.469* **(0.156)**
Period 18–20	**0.268** *** **(0.043)**	−0.064 (0.057)	0.299*** **(0.057)**	−0.228 (0.194)
Period 19–21	0.311*** **(0.040)**	0.004 (0.101)	**0.313** *** **(0.043)**	−0.113 (0.271)
Period 20–22	0.159*** **(0.047)**	**−0.245^.^ (0.126)**	0.187*** **(0.051)**	−0.322 (0.261)
Post-vaccination titer	**0.020** *** **(0.003)**	0.027*** **(0.007)**	0.016*** **(0.003)**	0.028 (0.016)
Moderate Boost level *(> 4 & ≤ 8)*	0.113*** **(0.028)**	**0.091^.^ (0.054)**	0.133*** **(0.033)**	0.137 (0.159)
High Boost level *(> 8)*	0.282*** **(0.028)**	0.278*** **(0.058)**	0.292*** **(0.034)**	0.348 (0.218)

1 measured in years

2 measured in kg/m^2^

(P-values were derived from Satterthwaite approximation

‘***’: *p* < 0.001

‘**’: *p* < 0.01

‘*’: *p* < 0.05, ‘.’: *p* < 0.1)

**Table 5:** Coefficient estimates (standard errors in parentheses; significance levels indicated by asterisk) of demographic factors from the linear mixed-effects model for each strain.

	H1N1	H3N2	Yamagata	Victoria
Age^1^	**0.001** *** **(0.000)**	**0.001** *** **(0.000)**	**0.001** * **(0.001)**	**0.001^.^ (0.000)**
Sex (Male)	−0.000 (0.009)	−0.004 (0.001)	0.008 (0.008)	0.005 (0.011)
BMI^2^	0.001 (0.001)	0.002* **(0.001)**	0.000 (0.001)	0.001 (0.001)
Prior vaccination (Yes)	0.020 (0.013)	0.008 (0.013)	**−0.020^.^ (0.010)**	0.004 (0.013)
Number of years participation	−0.006 (0.006)	0.001 (0.006)	0.006 (0.005)	0.000 (0.006)
Period 17–19	0.011 (0.017)	0.094*** **(0.018)**	0.126*** **(0.014)**	**0.167** *** **(0.017)**
Period 18–20	0.083*** **(0.017)**	**0.192** *** **(0.019)**	0.017 (0.015)	0.012 (0.019)
Period 19–21	0.132*** **(0.017)**	0.082*** **(0.017)**	0.018 (0.015)	0.087*** **(0.018)**
Period 20–22	0.039* **(0.020)**	0.075*** **(0.020)**	**0.034^.^ (0.018)**	0.016 (0.022)
Post-vaccination titer	0.007*** **(0.001)**	0.005*** **(0.001)**	0.003* **(0.001)**	0.006*** **(0.001)**
Moderate Boost level *(> 4 & ≤ 8)*	0.041*** **(0.012)**	0.003** **(0.012)**	**0.018^.^ (0.010)**	0.015 (0.012)
High Boost level *(> 8)*	0.082*** **(0.012)**	0.069*** **(0.012)**	0.066*** **(0.010)**	0.056*** **(0.013)**

1 measured in years

2 measured in kg/m^2^

(P-values were derived from Satterthwaite approximation

‘***’: p < 0.001

‘**’: p < 0.01

‘*’: p < 0.05, ‘.’: p < 0.1)

## Data Availability

The authors do not have permission to share data.

## Appendix A. Supplementary data

Supplementary data to this article can be found online at https://doi.org/10.1016/j.vaccine.2025.127904.
