# Supplementary material for: Assessing factors associated with one-year antibody waning in participants with repeated influenza vaccinations: A six-year cohort study
Source: Vaccine. Author manuscript; Available in PMC 2026 Jun 30. (PMC13317810; doi:10.1016/j.vaccine.2025.127904)
Supplement: 1 [file NIHMS2185401-supplement-1.docx]

**Supplementary Materials**

Table S1. average of log2-transformed HAI titers at pre-vaccination, post-vaccination in each influenza season

|  | **H1N1** | **H3N2** | **Yamagata** | **Victoria** |
| --- | --- | --- | --- | --- |
| 2016-17 pre-vac titer | 5.754 | 5.202 | 6.202 | 3.978 |
| 2016-17 post-vac titer | 7.918 | 7.560 | 8.396 | 7.411 |
| 2017-18 pre-vac titer | 5.917 | 6.734 | 6.758 | 6.075 |
| 2017-18 post-vac titer | 7.480 | 7.687 | 7.972 | 7.464 |
| 2018-19 pre-vac titer | 6.321 | 6.151 | 5.401 | 4.691 |
| 2018-19 post-vac titer | 7.701 | 7.241 | 6.701 | 6.141 |
| 2019-20 pre-vac titer | 4.875 | 3.699 | 5.365 | 5.145 |
| 2019-20 post-vac titer | 6.158 | 6.353 | 6.604 | 6.328 |
| 2020-21 pre-vac titer | 4.147 | 5.390 | 5.844 | 4.784 |
| 2020-21 post-vac titer | 5.549 | 6.723 | 6.549 | 5.897 |

Table S2. Chi-square test between Teenager and Adult in Sex difference

|  | **17-19** | **18-20** | **19-21** | **20-22** |
| --- | --- | --- | --- | --- |
| p-value | 0.539 | 0.146 | 0.675 | 0.317 |

Table S3. Coefficient estimates (95% confidence interval in parentheses; significance levels indicated by asterisk) of demographic factors from the linear mixed-effects model for overall samples and each age group.

|  | All  (n=584) | Teenagers (n=172) | Adults (n=386) | Elderly (n=26) |
| --- | --- | --- | --- | --- |
| Age | ${\boldsymbol{0.}\boldsymbol{004}}^{\boldsymbol{***}}$**(0.002, 0.005)** | **-**${\boldsymbol{0.}\boldsymbol{039}}^{\boldsymbol{*}}$**(-0.073, -0.004)** | ${\boldsymbol{0.}\boldsymbol{002}}^{\boldsymbol{*}}$**(0.000, 0.004)** | -0.009  (-0.034, 0.015) |
| Sex (Male) | -0.019  (-0.061, 0.023) | 0.023  (-0.061, 0.108) | -0.036  (-0.085, 0.012) | -0.091  (-0.277, 0.094) |
| BMI | ${\boldsymbol{0.}\boldsymbol{004}}^{\boldsymbol{*}}$**(0.000, 0.006)** | ${\boldsymbol{0.}\boldsymbol{012}}^{\boldsymbol{*}}$**(0.001,**  **0.021)** | 0.001  (-0.001, 0.005) | -0.002  (-0.018, 0.014) |
| Prior vaccination (Yes) | 0.016  (-0.040, 0.074) | -0.102  (-0.273, 0.079) | -0.004  (-0.069, 0.062) | **-**$\boldsymbol{0.477}^{\boldsymbol{.}}$  **(-0.825, -0.128)** |
| Number of years participation | -0.002  (-0.028, 0.024) | 0.104  (-0.013, 0.221) | -0.006  (-0.034, 0.022) | 0.120  (-0.020, 0.261) |
| Period 17-19 | $\boldsymbol{0.395}^{\boldsymbol{***}}$ **(0.318, 0.472)** |  | ${\boldsymbol{0.4}\boldsymbol{17}}^{\boldsymbol{**}\boldsymbol{*}}$ **(0.329, 0.507)** | ${\boldsymbol{0.4}\boldsymbol{69}}^{\boldsymbol{*}}$ **(0.243, 0.693)** |
| Period 18-20 | $\boldsymbol{0.268}^{\boldsymbol{***}}$**(0.182, 0.351)** | -0.064  (-0.177, 0.046) | ${\boldsymbol{0.2}\boldsymbol{99}}^{\boldsymbol{***}}$ **(0.186, 0.409)** | -0.228 (-0.506, 0.051) |
| Period 19-21 | ${\boldsymbol{0}\boldsymbol{.311}}^{\boldsymbol{***}}$ **(0.229, 0.390)** | 0.004  (-0.205, 0.193) | ${\boldsymbol{0}\boldsymbol{.313}}^{\boldsymbol{***}}$**(0.225, 0.399)** | -0.113 (-0.502, 0.277) |
| Period 20-22 | ${\boldsymbol{0}\boldsymbol{.159}}^{\boldsymbol{***}}$ **(0.064, 0.250)** | **-**$\boldsymbol{0.245}^{\boldsymbol{.}}$  **(-0.489, 0.002)** | ${\boldsymbol{0}\boldsymbol{.187}}^{\boldsymbol{***}}$ **(0.085, 0.287)** | -0.322 (-0.698, 0.053) |
| Post-vaccination titer | ${\boldsymbol{0.}\boldsymbol{020}}^{\boldsymbol{***}}$**(0.012, 0.026)** | $\boldsymbol{0.027}^{\boldsymbol{***}}$ **(0.010,**  **0.043)** | $\boldsymbol{0.016}^{\boldsymbol{***}}$ **(0.008, 0.023)** | 0.028 (0.004, 0.051) |
| Moderate Boost level | $\boldsymbol{0.113}^{\boldsymbol{***}}$ **(0.056, 0.171)** | $\boldsymbol{0.091}^{\boldsymbol{.}}$  **(-0.020, 0.215)** | $\boldsymbol{0.133}^{\boldsymbol{***}}$ **(0.068, 0.201)** | 0.137 (-0.091, 0.365) |
| High Boost level | $\boldsymbol{0.282}^{\boldsymbol{***}}$ **(0.217, 0.351)** | $\boldsymbol{0.278}^{\boldsymbol{***}}$ **(0.133,**  **0.431)** | $\boldsymbol{0.292}^{\boldsymbol{***}}$ **(0.218, 0.365)** | 0.348 (0.035, 0.661) |

(P-values were derived from Satterthwaite approximation; ‘***’: p<0.001, ‘**’: p< 0.01, ‘*’: p< 0.05, ‘.’: p<0.1)

Table S4. Coefficient estimates (95% confidence interval in parentheses; significance levels indicated by asterisk) of demographic factors from the linear mixed-effects model for overall samples and each age group from samples with negative waning scores.

|  | All  (n=802) | Teenagers (n=244) | Adults (n=522) | Elderly (n=36) |
| --- | --- | --- | --- | --- |
| Age | ${\boldsymbol{0.}\boldsymbol{005}}^{\boldsymbol{***}}$**(0.004, 0.007)** | -0.016  (-0.049, 0.016) | ${\boldsymbol{0.}\boldsymbol{003}}^{\boldsymbol{**}}$**(0.001, 0.005)** | 0.001  (-0.035, 0.038) |
| Sex (Male) | -0.001  (-0.044, 0.042) | $\boldsymbol{0.077}^{\boldsymbol{.}}$  **(-0.008, 0.163)** | -0.031  (-0.080, 0.019) | -0.023  (-0.223, 0.177) |
| BMI | ${\boldsymbol{0.}\boldsymbol{003}}^{\boldsymbol{*}}$**(-0.000, 0.007)** | 0.004  (-0.005, 0.014) | 0.002  (-0.002, 0.006) | -0.004  (-0.024, 0.017) |
| Prior vaccination (Yes) | 0.022  (-0.033, 0.078) | **-**$\boldsymbol{0.114}^{\boldsymbol{.}}$  **(-0.242, 0.017)** | -0.003  (-0.071, 0.065) | **-**$\boldsymbol{0.338}^{\boldsymbol{.}}$  **(-0.672,**  **-0.040)** |
| Number of years participation | 0.005  (-0.020, 0.030) | 0.051  (-0.054, 0.156) | -0.003  (-0.029, 0.024) | 0.115  (0.001, 0.238) |
| Period 17-19 | $\boldsymbol{0.482}^{\boldsymbol{***}}$ **(0.409, 0.556)** |  | ${\boldsymbol{0.5}\boldsymbol{38}}^{\boldsymbol{**}\boldsymbol{*}}$ **(0.450, 0.629)** | ${\boldsymbol{0.7}\boldsymbol{18}}^{\boldsymbol{***}}$ **(0.466, 0.941)** |
| Period 18-20 | ${\boldsymbol{0.3}\boldsymbol{48}}^{\boldsymbol{***}}$**(0.269, 0.424)** | -0.033  (-0.147, 0.076) | ${\boldsymbol{0.4}\boldsymbol{26}}^{\boldsymbol{***}}$ **(0.317, 0.531)** | 0.196 (-0.148, 0.475) |
| Period 19-21 | ${\boldsymbol{0}\boldsymbol{.358}}^{\boldsymbol{***}}$ **(0.279, 0.433)** | 0.115  (-0.062, 0.292) | ${\boldsymbol{0}\boldsymbol{.336}}^{\boldsymbol{***}}$**(0.248, 0.419)** | 0.243 (-0.081, 0.565) |
| Period 20-22 | ${\boldsymbol{0}\boldsymbol{.262}}^{\boldsymbol{***}}$ **(0.172, 0.351)** | -0.120  (-0.357, 0.123) | ${\boldsymbol{0}\boldsymbol{.303}}^{\boldsymbol{***}}$ **(0.204, 0.398)** | 0.113 (-0.265, 0.486) |
| Post-vaccination titer | ${\boldsymbol{0.}\boldsymbol{034}}^{\boldsymbol{***}}$**(0.026, 0.042)** | $\boldsymbol{0.049}^{\boldsymbol{***}}$ **(0.031,**  **0.064)** | $\boldsymbol{0.027}^{\boldsymbol{***}}$ **(0.019, 0.035)** | $\boldsymbol{0.051}^{\boldsymbol{**}}$ **(0.020,**  **0.076)** |
| Moderate Boost level | $\boldsymbol{0.123}^{\boldsymbol{***}}$ **(0.067, 0.185)** | 0.050  (-0.051, 0.173) | $\boldsymbol{0.157}^{\boldsymbol{***}}$ **(0.090, 0.228)** | 0.160 (-0.038, 0.375) |
| High Boost level | $\boldsymbol{0.267}^{\boldsymbol{***}}$ **(0.200, 0.340)** | $\boldsymbol{0.148}^{\boldsymbol{**}}$ **(0.032,**  **0.292)** | $\boldsymbol{0.330}^{\boldsymbol{***}}$ **(0.253, 0.411)** | $\boldsymbol{0.430}^{\boldsymbol{*}}$ **(0.119,**  **0.729)** |

(P-values were derived from Satterthwaite approximation; ‘***’: p<0.001, ‘**’: p< 0.01, ‘*’: p< 0.05, ‘.’: p<0.1)
